# Automated forest land division using deep learning and drone imagery

**DOI:** 10.1371/journal.pone.0335009

**Published:** 2025-10-31

**Authors:** Kushagra Umesh Borse, Ninad Nilesh Sugandhi, Chirayu Batra, Maria Anu Vensuslaus

**Affiliations:** School of Computer Science and Engineering, Vellore Institute of Technology, Chennai, India; University of Maryland at College Park, UNITED STATES OF AMERICA

## Abstract

This paper proposes an automated solution for tree enumeration in areas designated for forest land division using drone image processing. Traditional tree counting methods are time-consuming and error-prone. Our approach leverages drone imagery and advanced computer vision algorithms. The solution demonstrates the potential to accurately detect tree crowns, facilitating informed decision-making in forest land division projects, promoting sustainability and efficient resource management.

## Introduction

Forests play a crucial role in global ecosystems but are increasingly being diverted for development projects, requiring accurate tree enumeration to perform sustainable land management. Traditional methods of tree counting, such as manual surveys, are labor-intensive, costly, and prone to errors. These limitations highlight the need for more efficient, automated solutions. This research proposes an advanced approach for tree enumeration, utilizing drone technology and computer vision algorithms to automate the process. Drones equipped with high-resolution cameras can capture detailed imagery over vast and challenging terrains, enabling precise tree detection and categorization.

The goal of this study is to design and validate an automated tree enumeration system to improve the efficiency and accuracy of tree data collection, supporting more informed decisions in forest land division projects. By addressing key aspects such as image data analysis, tree counting, scalability, and ethical considerations, this approach aims to provide a practical, scalable solution for sustainable forest management in the face of growing urbanization.

This research drew its data from various public and private sources which included government agencies together with environmental research organizations and open satellite imagery databases. Images were obtained at various pixel dimensions which depended upon the data provider’s specifications while satellite imagery gained resolutions between 1 to 5 meters per pixel yet drone imagery exhibited sub-10 cm pixel resolution levels. A set of preprocessing operations was utilized to allocate geographic coordinates, reduce noise signals and improve brightness contrast then unify image parameters throughout the different data sets. The selected model resolution settlement stood at 512 × 512 pixels because it managed to produce optimal detection results without unnecessary computer processing wastes.

The developed method leads to direct applications in environmental monitoring and policy development. Numerous government agencies can put this approach to work when planning extensive reforestation activities and when assessing urban greenery areas and protecting biodiversity. The inclusion of computerized tree counting capabilities in regulatory standards enables authorities to execute forest protection regulations while conducting environmental law monitoring. Using this system enables infrastructure projects to evaluate environmental effects thus facilitating better implementation of ecological replacement strategies. Through measurements of tree quantity and layout policymakers gain the ability to make decisions for zoning policies along with conservation plans and carbon reduction programs which connect technological insights with sustainable leadership practices.

The described methods are scalable and adaptable for analyzing both large and small areas, depending on the imagery source. Satellite data is particularly effective for large-scale environmental assessments, while drone imagery provides detailed insights for localized regions. This flexibility makes the methodology suitable for diverse spatial scales and project requirements.

### Related works

The rapid expansion of urbanization and infrastructure projects has led to significant forest land division, necessitating accurate and efficient tree enumeration methods. Drone technology has emerged as a key tool in automating data collection for this purpose, providing flexibility, ease of operation, and cost-effectiveness. This literature review explores advancements in drone-based automated tree enumeration, emphasizing innovations in image processing for enhanced accuracy and efficiency.

### Tree detection techniques

Convolutional Neural Networks succeed in detecting and classifying trees when applied to satellite imagery analysis. The identification of tree patterns at scale is a strong point of CNN-based models because they leverage deep feature extraction to enhance detection accuracy according to [1,2]. Drone imagery transition promotes new hurdles because image perspectives differences and resolution differences and changes in environmental conditions set limitations. Unlike satellite images, drone-captured data provides finer details but also requires more complex preprocessing and model adaptation.

### 3D data collection methods

Unmanned aerial vehicles (UAVs) capture detailed 3D data through two primary methods:

(1) LiDAR sensors, which penetrate canopies and generate comprehensive 3D data, and
(2) high-resolution RGB cameras that utilize Structure from Motion (SFM) to derive 3D canopy surface data [3].

Each approach has unique implications for individual tree segmentation, impacting accuracy based on environmental factors and image acquisition techniques.

### Machine learning in tree enumeration

Machine learning techniques, particularly deep neural networks, play crucial role in tree categorization. PointNet++ and KPConv, for example, have shown improved performance in segmenting 3D LiDAR data, especially with advanced data preparation techniques [4,5].

### Computational efficiency

For large-scale applications, computational efficiency is essential. Studies on parallel processing have shown that processing times for extensive drone imagery datasets can be significantly reduced, making real-time tree enumeration feasible for large forested regions [6]. However, the trade-off between model complexity and inference speed remains a challenge, particularly when processing ultra-high-resolution drone images.

### Image resolution and accuracy

Image resolution is another critical factor, influencing the accuracy of vegetation classification and segmentation, as higher resolutions facilitate better delineation of individual species [7]. Automated tree enumeration requires rigorous validation to ensure accuracy. Therefore, selecting an optimal resolution is a balance between computational feasibility and classification accuracy.

### Validation and ethical considerations

Cross-referencing automated outputs with ground-truth data enhances reliability, minimizing false detections [8]. As these systems advance, ethical considerations, such as data privacy and environmental compliance, gain importance, with studies suggesting best practices for responsible deployment [9].

### Dataset preparation and preprocessing

The custom dataset consists of images of banana trees, oil palm trees, and coconut trees. The datasets were organized and annotated on Roboflow, with quality checks to address any mislabeling, ensuring a reliable training dataset. Model training considered factors like epochs, image size, and dataset specifications, chosen due to YOLOv8’s efficiency in object detection tasks. The selected tree species possess economic value because of their widespread cultivation throughout tropical and subtropical regions. The need for accurate classification explains why these tree species are appropriate for automated detection because they serve agricultural monitoring needs and yield estimation as well as sustainable land use planning.

To facilitate accurate classification of banana, oil palm and coconut trees, the dataset for this project was accumulated from various databases and repositories to include different environments, developmental phases, and angles of observations. This diverse dataset ensures the inclusion of different environments, developmental phases, and varying observational angles, improving model robustness. All the images were labeled through a rigorous procedure made possible using Roboflow. To eliminate any noise in the labeling, quality checks were conducted on the dataset to identify and eliminate any mislabeling in the data. The validation of this process was done in parallel with automated checks and manual inspection to ensure the correctness of class labels and bounds of the boxes. The images in the dataset were captured at different resolutions ranging from 800x600 to 1200x800 pixels, which was inconsistent. To make it uniform, all images were resized to a consistent resolution of 512x512 pixels. The resolution level was picked to maintain optimal computational speed and retain necessary visual information needed for accurate species identification. The selected resolution reaches a balance between computational efficiency and structural feature preservation which is vital for separating tree species from the images. Standardizing the resolution allows the model to process data in a consistent format, ensuring that it can learn the features effectively without being affected by varying image sizes.

In the case of normalization, it normalized all the values of pixels into a range lying between 0 and 1. This is achieved by dividing the pixel values by 255 since typical pixel values generally lie between 0 and 255 in an image. This min-max normalization ensures that the input values fall within a range more suitable for training neural networks. Normalizing in this manner ensures that the model focuses on the learning of appropriate features and avoids the impact of large pixel value variations. Thus, the training converges much more efficiently.

The dataset consisted of images of varying environmental conditions. The pixel resolution in the images equates to real-world coverage; that is, 1 pixel = 1 square meter. This is information whose clarity is pertinent because it relates to describing the scope or scale of the data set. Geographical coverage of the dataset will also be useful to assess diversity and relevance for training data. The model would then be capable of handling various scenarios, including different terrains or environmental conditions.

Ground-truth data were manually annotated or obtained through automated approaches for validation; this ensured label accuracy. In the case of this study, a 90%−10% data split was employed, with 90% used for training and 10% for validation. This split was chosen over conventional 80%−20% or 70%−30% distributions to maximize training data while still retaining a sufficient portion for validation. A larger training set helps the model learn complex patterns more effectively without overfitting. The cross-validation can be used to further enhance the validation process, especially if the dataset is smaller, as it would allow for more robust performance evaluation. In addition to this, the performance metrics used for validation included precision, recall, and F1 score. For object detection tasks, Intersection over Union (IoU) was also calculated to assess the accuracy of predicted bounding boxes, ensuring the model’s reliability.

## Method

### Data preprocessing and annotation

Aerial photos and related shapefiles served as direct inputs for annotation as well as generation of training data. Shapefiles adopted in this research were point shapefiles, in which every point was considered the geographical location of a single tree (i.e., coconut, oil palm, or banana). These point geometries were retrieved and transformed into geoJSON objects to facilitate easier integration with image annotation tools.

For every configured geoJSON object, we retrieved coordinate pairs (latitude, longitude) and applied affine transformation methods to project these geolocations onto their respective bounding pixel locations on the aerial photos. The points thus configured were utilized to create bounding boxes around observable tree crowns.

Annotation was done in Roboflow, where bounding boxes were manually inspected and corrected. This combined annotation method (automated + manual QA) assisted in maintaining high label accuracy. All annotations were also standardized into the YOLO annotation format (class, x_center, y_center, width, height) to ensure consistency among detection models.

### Dataset standardization

Images generated from different drones and databases ranged from resolutions of 800 × 600 to 1200 × 800 pixels. All were resized to a default resolution of 512 × 512 pixels, which was selected based on its balance between detection accuracy and computational complexity.

Pixel values were normalized to the range [0, 1] by dividing each pixel’s intensity by 255. Normalization decreased training instability and brought consistency across training batches.

For dealing with lighting and shadowing variations, histogram equalization and contrast adjustment were used as preprocessing. These operations enhanced visual contrast between tree crowns and background ground, which aided downstream model performance.

### Aerial Eye Tree Detection Algorithm (AETDA)

The Aerial Eye Tree Detection Algorithm (AETDA) starts with preprocessing aerial images and shapefiles to create training and validation datasets. Key preprocessing steps involve extracting the geographical coordinates of coconut, oil palm, and banana trees from geoJSON objects across multiple datasets, resulting in pixel-mapped images with annotated tree locations.

For this purpose, in the present work, different deep learning architectures were employed to detect and classify banana, oil palm, and coconut trees from the images. The evaluated models are ResNet, VGG16 with a detection head, DETR, and YOLOv8. In this task, each model had its advantages and disadvantages in its usage.

Below is a breakdown of the comparison made as follows.

#### 1. ResNet.

Among the algorithms, ResNet was chosen due to its feature extraction feature and high accuracy in image classification as a prediction algorithm. But ResNet operates as a classifier without the inclusion of the object detection mode. Hence, while it was able to give reasonable classification accuracy when only one tree was present, it could not localize several trees in an image or classify several tree types at the same time. This restriction rendered ResNet unsuitable for the tasks that require precise spatial identification together with multi-instance annotation, which are the primary constraints in the dataset. Moreover, ResNet used global image-level features instead of the localized object features for image recognition, and it imposed more requirements on ResNet for the project objectives.

#### 2. VGG16 with detection head integration.

To overcome the limitations of ResNet, the VGG16 was used conjugated with another detection head. Therefore, it extracts the detailed feature maps of the input images using the not very deep but complex VGG16 network. This detection head that has been suggested in the research is able to assess these feature maps, generate the bounding box, and estimate the class labels for the purpose of detection as well as classification. This methodology introduced advancements over the ResNet because it can detect multiple objects within the same image and identify the location as well as the type of trees in an image. However, even with these enhancements, VGG16 failed to receive satisfactory bounding box precision coupled with accurate class prediction. Also, its computational requirements were high, and, when training the model, the model exhibited relatively slow convergence.

#### 3. DEtection TRansformer DETR.

DETR was then chosen to benefit from the transformer architecture for object detection tasks. DET does not rely on convolutional heads but utilizes self-attention to learn the spatial relation and detect and classify multiple instances. DETR was adjusted to generate the output corresponding to the given dataset. DETR demonstrated progress in determining the correct number of trees and made good estimations of the types of trees. However, there were problems with the annotation process of DETR. For as much as it was effective in localizing objects in the images, the quality of the bounding box as well as the class labels sometimes contained mistakes. This misalignment often led to problems in creating high-precision annotations that are core in the study. This also affected the training of DETR, which had high computational demands resulting from the architecture proposed by the authors. Consequently, while showing excellent results in increasing multi-object detections on the test set compared to ResNet and VGG16, DETR could not provide stable annotations as it should have.

### YOLOv8

Since there were many difficulties that were observed while using the previous models, YOLOv8 was utilized in the last instance. Since YOLO is an improved version of the YOLO (You Only Look Once) architecture, it is optimized for real-time object detection by default. It provides a proper balance of architecture that is conducive to both the time taken by the inference as well as the localization. Getting accurate bounding box annotations, high-class prediction, and efficient multi-object detection, YOLOv8 surpassed the previous models.

For training, a dataset of such mapped images is then fine-tuned on pre-trained YOLO model weights as needed to optimize the model to the characteristics of aerial imagery. This selective fine-tuning enables the model to be fine-tuned for aerial images while at the same time exploiting prior knowledge.

The advantages of YOLOv8 are considerably worth mentioning. Since it is a lightweight and efficient detector, it takes very little time to detect, classify, and annotate as many trees as possible in one go. Through techniques such as anchor boxes and Non-Maximum Suppression (NMS), we can get rid of the duplicate bounding boxes and improve the precision of detection at a negligible additional price. The results of the experiments showed that YOLOv8 outperformed the original DETR on most of the metrics we assessed, including IoU annotation accuracy, which was considerably higher and more consistent in this task, and since YOLOv8 was able to meet the localization and classification requirements of this dataset, it proved to be ideal for this project.

**Fig 1:**
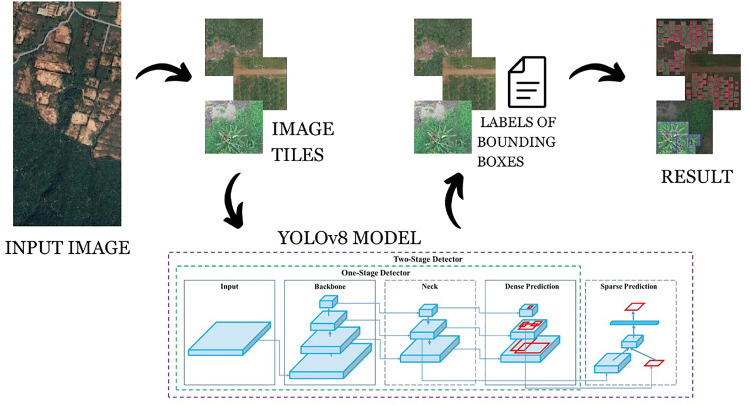
AETDA architecture diagram.

The YOLOv8 [10] model architecture illustrated in Fig 1 comprises the following: The backbone takes a convolutional neural network (CNN) to extract features of the input images. The Neck sub-module is used to handle the problem of the up-sampling and down-sampling of the features to handle the shortcomings of the features in representation. The detection head outputs positions of the objects in the image and decides the type of the objects, and the last stage is confined by non-max suppression to only a few bounding boxes.

YOLO (You Only Look Once) is a widely used object detection model known for its real-time performance. YOLOv8, released on January 10^th^, 2023, incorporates a Path Aggregation Network (PANet) for enhanced feature fusion and a new detection head for improved accuracy and speed. With 225 layers and over 3 million parameters, YOLOv8 effectively handles the tree classification task, utilizing its backbone for feature extraction and head for producing bounding boxes and class labels across different scales.

## Results

The metrics used in the assessment of an object detection model are very significant since they offer insight into the performance of a given model. Box(P) (Precision) represents the accuracy as the ratio of the true positives to the total number of positive predictions. The Box(R) (Recall) represents the completeness as the proportion of the actual positive that has been correctly identified. The mAP50 of the calculated metric is the mean average precision at an Intersection over Union (IoU) threshold of 0.5, which gave a balance between precision and recall. On the other hand, mAP50-95 is calculated as the arithmetic mean of AP scores under different IoU thresholds between 0.5 and 0.95, which will present a more generalized evaluation of the model.

**Fig 2:**
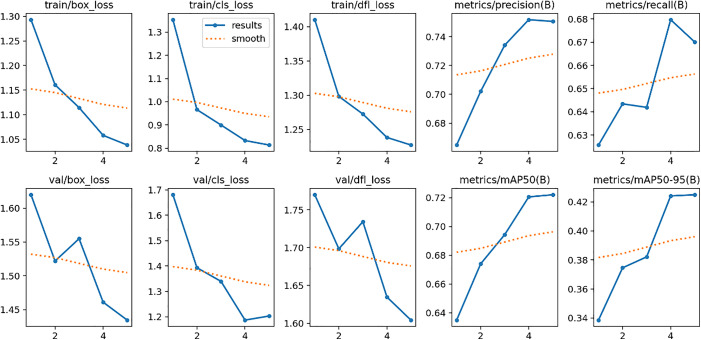
Performance metrics of the proposed tree classification model on the training dataset.

Table 1 summarizes these metrics, with Fig 2 visualizing the model’s training performance, demonstrating low loss and high precision, recall, and mAP scores, indicating effective tree classification.

**Table 1:** Summary table for method section.

Method	Resolution	Cost	Data volume	Preprocessing	Accuracy	Inference Speed
Satellite CNN	1-10 m/ pixel	Low	Low	Moderate	Moderate	High
Drone CNN	Sub 10 cm	Moderate	Medium	High	High	High
LIDAR Pointnet	Few cm	High	High	Very high	Very high	Low
SfM 3D segmentation	Few cm	Moderate	Medium	High	High	Medium

Table 2 captures the classification accuracy and error distribution for tree detection: It shows the summary of the model’s detection performance for coconut, oil palm, and banana trees. True Positives (TP) of the classes were 270, 7393, and 1093, respectively, meaning correctly classified cases. False Positives (FP) were 44, 2027, and 447, meaning over-predicted cases. False negatives were 296, 868, and 483, which meant missed cases. True negatives were 12354, 2632, and 10897, which corresponded to correct rejected cases. The model effectively detects tree classes but requires optimization to minimize false positives and negatives for improved accuracy.

**Table 2:** Evaluation metrics.

Class	Image	Instance	Box (P)	Box (R)	mAP50	mAP50-95
**ALL**	3579	10403	0.751	0.67	0.722	0.425
**BANANA-PLANT**	1193	1576	0.71	0.662	0.702	0.34
**OIL** **PALM** **TREE**	1193	8261	0.792	0.882	0.912	0.632
**COCONUT** **TREE**	1193	566	0.75	0.466	0.552	0.303

The confusion matrix depicted in Fig 5 below further illustrates predictive accuracy. The diagonal elements give correct predictions where the model predicts 270 coconut trees, 7393 oil palm trees, and 1093 banana trees correctly. Off-diagonal elements denote incorrect predictions, such as one banana tree being misclassified as a coconut tree. These insights indicate a high discriminative capacity of the model between the classes as well as the areas of improvement.

**Fig 3:**
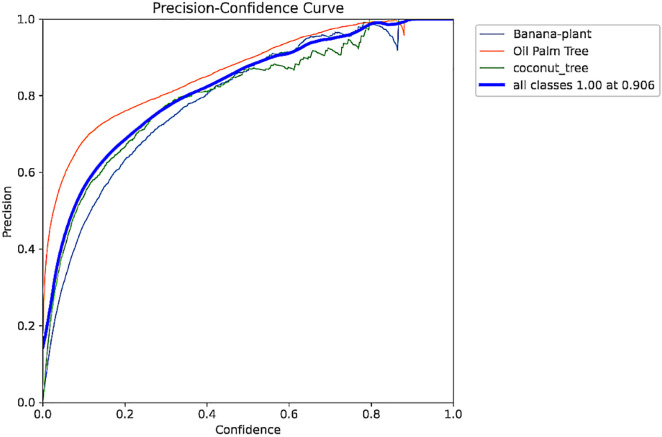
Precision curve.

**Fig 4:**
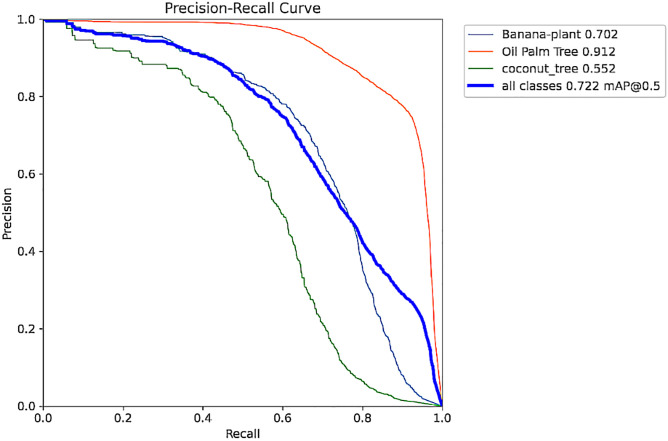
Precision Recall curve.

The Precision Curve in Fig 3 shows the precision against varying confidence levels. The high accuracy of the model is evident through this curve, which shows that the model maintains a high level of precision at different thresholds, meaning even at lower levels of confidence, the trees can be well detected. This allows users to pick the best threshold that returns high precision without a considerable loss of recall. In the same manner, Fig 4 shows the Precision-Recall (PR) Curve to illustrate the precision against recall values at each threshold. The AUPRC of the PR curve of 0.95 indicates that the proposed model yields high precision and the effective recall of tree classes.

**Fig 5:**
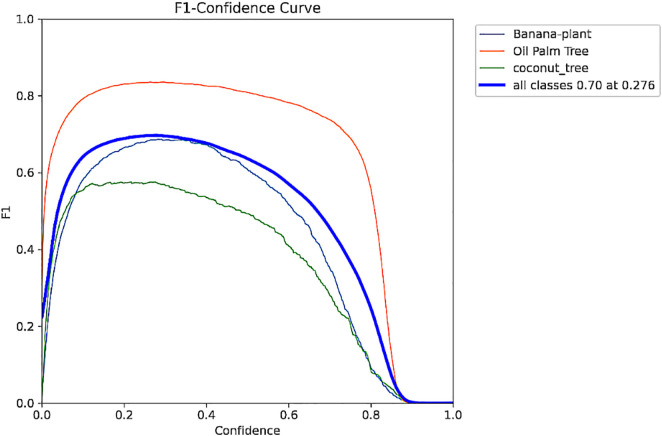
F1 Confidence curve.

**Fig 6:**
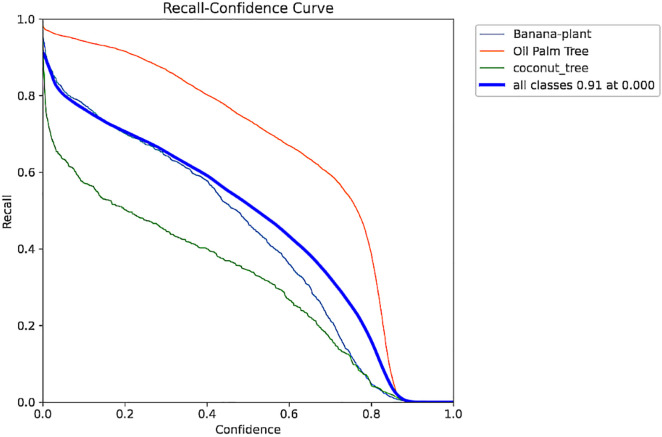
Recall Confidence curve.

The F1-Confidence Curve represented in Fig 5 shows the variation of F1-Score with respect to varying confidence levels. F1-score is the harmonic mean of precision and recall. This curve shows how the F1 score remains high; hence, it can balance between precision and recall throughout the model. These kinds of visualizations help when defining the thresholds that provide the most favorable performance characteristics. Also, the Recall-Confidence Curve in Fig 6 relates recall and confidence thresholds; as the former rises, the latter typically drops, while precision decreases. This phenomenon is backed up by Table 3, which shows that lower thresholds equal high recall and relatively low precision. It is therefore possible to iterate on these trends to get the right thresholds for the right recall and precision for greater utility of this model.

**Table 3:** Confusion matrix.

Class	True Positives (TP)	False Positives (FP)	True Negatives (TN)	False Negatives (FN)
Coconut	270	44	12354	296
Oil Palm	7393	2027	2632	868
Banana	1093	447	10897	483

**Fig 7:**
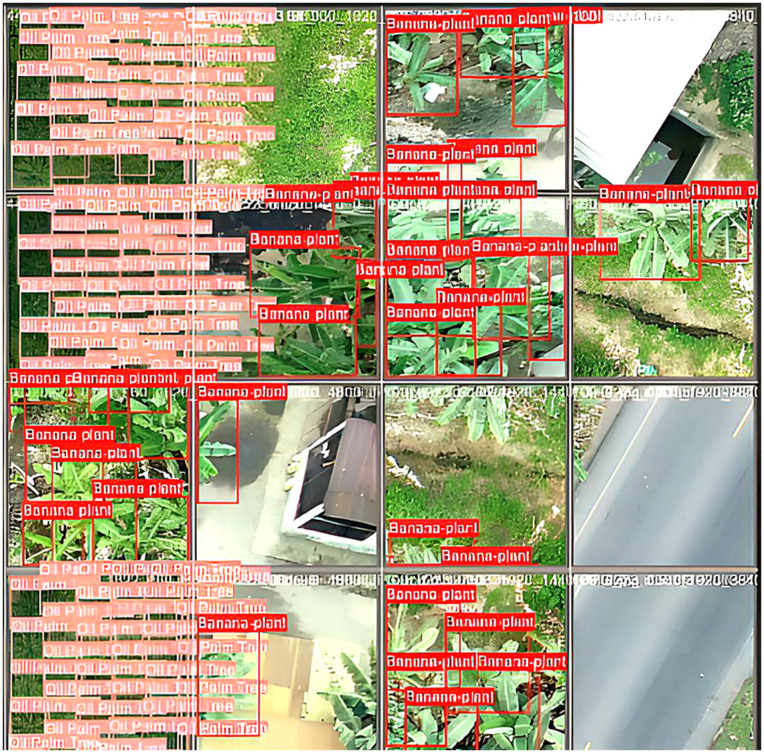
Annotations and predictions on several testing images containing different trees.

Fig 7 displays a high-resolution aerial image of a tropical forest, annotated with ground truth bounding boxes for coconut, oil palm, and banana trees. Predicted bounding boxes overlay these annotations, showing the model’s accurate localization and classification of each tree type. No misclassifications were observed, indicating the model’s precision in differentiating between species.

### presented in YOLO results

**Fig 8:**
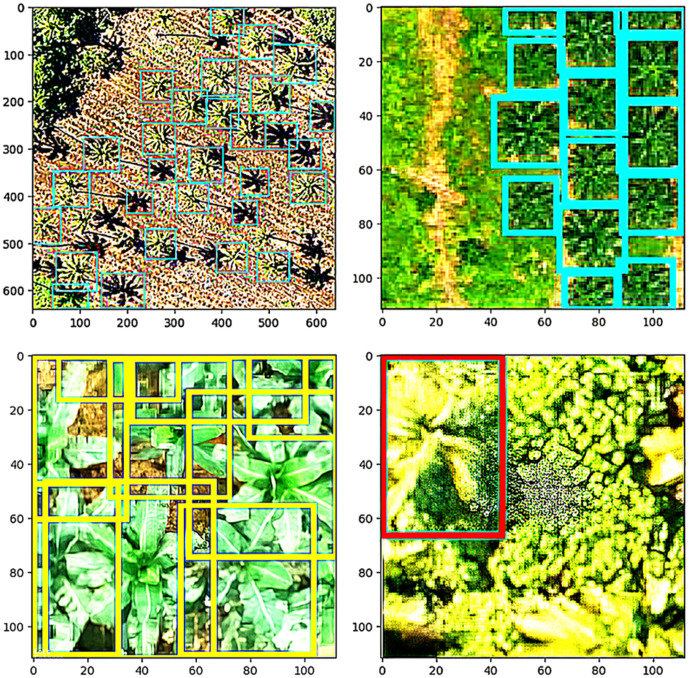
VGG16 Tree Classification Model Visualization on diverse tree types.

Fig 8 highlights the VGG16 model’s capacity to identify and categorize diverse tree types with clear bounding boxes and labels in high-resolution images. Each tree type is outlined in distinct colors: yellow for banana, cyan for oil palm, and red for coconut, enhancing visual clarity and demonstrating precise classification as shown in Tables 5 and 6.

**Table 4:** Effect of confidence threshold on recall and precision.

Confidence Threshold	Recall	Precision
0.95	0.90	0.98
0.90	0.95	0.93
0.85	0.97	0.88

**Table 5:** Performance metrics comparison of VGG16 model with detection head.

Metric	Epoch 1	Epoch 5	Epoch 10
Training Accuracy	88.2%	98.95%	99.71%
Training Loss	0.3156	0.2756	0.2580
Validation Accuracy	96.57%	96.74%	96.94%
Validation Loss	0.3460	0.3394	0.3302
Final Test Accuracy	98.39%		

**Table 6:** Performance metrics comparison of VGG16 model with detection head, YOLOv8, DETR, ResNet.

Model	Precision	Recall	mAP@0.5	mAP@0.5:0.95	Inference Time	Bounding Box Accuracy	Notes
YOLOv8	0.751	0.670	0.722	0.425	Fast	High	Best all-around performer
DETR	0.703	0.633	0.681	0.382	Moderate	Moderate (Edge misses)	Strong counting, weaker localization
VGG16 + Detection Head	0.695	0.620	0.668	0.358	Slow	Moderate	Strong classification, less precise detection
ResNet	0.88 (classification only)	N/A	N/A	N/A	Very fast	Not applicable	No object detection functionality

The training and validation metrics for the VGG16 model with a detection head assessed in epochs 1, 5, and 10 are presented in the Table 4. From Table 4 it can be observed that the accuracy was increasing from epoch to epoch with the training accuracy of 99.71% in epoch 10 and the validation accuracy of 96.94% in epoch 10. Training loss in the same manner reduced from 0.3156 in Epoch 1 to 0.2580 in Epoch 10, which is a sign of improved model fit. Based on the above results, it can also be noted that validation loss was gradually decreasing, which means good generalization on new data. The obtained test accuracy of 98.39% also indicates effective classification and the ability of the model to identify multiple tree types in an image. This shows how the custom detection head works well, but there is still a small drawback in terms of localization compared to much more specialized detection models like YOLOv8.

**Fig 9:**
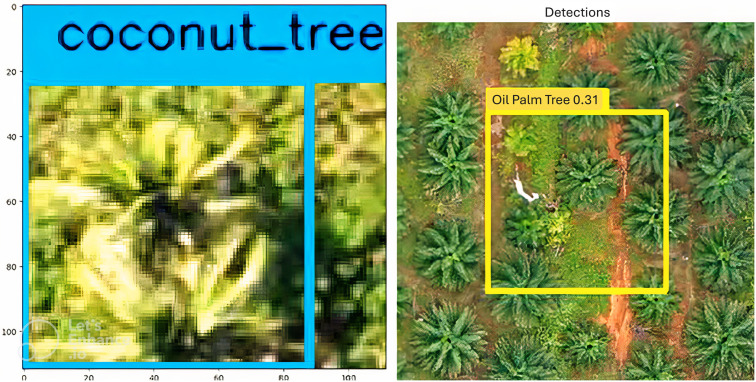
Annotations and predictions on testing images containing different trees presented in DETR results.

The DETR model tends to focus on the center of the image, as shown in Fig 9, effectively detecting trees located in that area but often missing trees around the edges, thereby overlooking finer details in the image. In contrast, VGG16 with a detection head performs better in capturing a broader range of tree locations across the entire image. While DETR can achieve accurate tree counts with adjustments to the confidence threshold, VGG16 provides more comprehensive coverage, enhancing overall detection quality.

**Fig 10:**
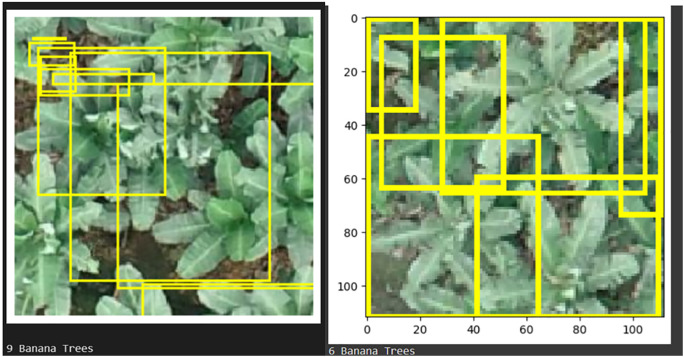
Model Predictions and Tree Count in a testing image.

Fig 10 presents the predicted bounding boxes and the tree count for a testing image, showcasing the model’s accuracy in both identifying tree types and quantifying their presence. This configuration underscores the model’s effectiveness in real-world, complex environments, supporting accurate and efficient tree classification in aerial imagery.

### Comparison with state-of-the-art method

Automated tree mapping using drones has become important in solving the environmental and operational issues arising from urbanization and deforestation. This means that the use of aerial imagery as a means of collecting data in a short time with high resolution has ensured their role in the field of forestry, agricultural planning, and the monitoring of the environment. There has been considerable work done in the past to use Convolutional Neural Networks (CNNs) for analyzing satellite images, and the results are extremely promising in the case of tree detection [1,2]. Yet, when it comes to using the same methodologies on aerial imagery, new issues arise, such as variable viewpoints, pixel densities, and intricate scenes [3]. In this area, deep learning models like PointNet++ and KPConv used for LiDAR data segmentation [4,5] as well as U-Net methods have been applied to individual tree crown delineation in high-resolution imagery [1], and.

Ensemble and hybrid models also enhance the field with a specialized combination of modalities or architectures to enhance species discrimination as well as segmentation accuracy [11–13]. For instance, Plesoianu et al. merge tree crown detection and classification into species in high resolution images [13] and Kouvaras & Petropoulos propose a method for detection and segmentation of trees from UAV images [12]. Zhao et al. presented use of multitemporal LiDAR data for forest biomass and carbon dynamics monitoring [14], whereas Leckie et al. presented initial fusion of LiDAR and multispectral data for crown level studies [15] of single trees. Machine learning has also been investigated by Cetin & Yastikli to classify urban tree species from the remote sensing data [16]. More generally, Zhao et al. propose a systematic review of individual tree crown methods based on CNNs [17] and Zhong et al. review remote sensing and deep learning strategy for tree species classification [11]. Rowan et al. describe the wider role of digital transformation in ecological systems like paludiculture and how remote sensing and digital methods can facilitate real-time sustainable monitoring [18]. In urban scenes, Ventura et al. utilize fine scale multispectral imagery and deep learning for large-scale tree detection proving the scalability of deep learning to dense urban scenes [19]. Liu’s research on multi-seasonal UAV multispectral approaches to tree species classification [20], Matejčíková et al.’s comparison of dead tree detection methods among data types [21], Atanasov et al. development of algorithm to detect white-flowering honey trees within mixed forests using UAV RGB imaging [22] and Zhang et al.’s combined modeling of canopy heights and images for single tree counting [23]. Recent works include Chen et al. for a zero-shot tree detection by means of pretrained segmentation models [Zero-Shot Tree Detection] and Gominski et al. for global-level large-scale tree mapping via Gaussian crown modeling [Trees as Gaussians].

Alongside progress in algorithms, close dataset design and benchmarking are also to be considered as a part of progress. Cases in point, LiDAR and imagery data used together over hundreds of square kilometers in the creation of the PureForest dataset is utilized in multimodal species classification. Meanwhile, the study by Zheng et al. provides a review on trends and problems of single tree crown detection from optical remote sensing, and summarizes different algorithm families and their application scenarios. The operations highlight the significance of innovation of methods and also the diversity of data. By placing the current work in the context of this broad literature with regard to classification/localization and efficiency, the proposed VGG16 + detection head model is an attempt to fill the gap between pure classification and full detection of objects, providing a viable compromise that is well-adapted to aerial imagery.

To tackle these difficulties in contrast to prior research, our study incorporates a new detection head to the VGG16. From being a pure classifier that differentiates between two classes of images it transforms the model into a model that can classify as well as localize. This approach is particularly strategic given that RGB-based aerial imagery has, for instance, more density canopy and various tree distributions that call for more dependable models [3,24]. The effectiveness of our model is evident in the results: The final test accuracy was 98.39%, proving its effectiveness, while the results obtained using traditional methods were considerably lower. In addition, the detection head contributed to object detection with the overall mAP50 of 0.722 across all the classes and class-specific results with the highest mAP50-95 of 0.632 for oil palm trees. These metrics highlight the fact that our model locates the object of interest with high accuracy while minimizing the computational time, which other studies that focused on classification or segmentation alone could not achieve [4,6].

One more advantage of the proposed approach is its ability to operate under any confidence level; recall and precision values indicate that the approach is quite resilient. For instance, at a confidence threshold of 0.90, the model got a recall of 0.95 and precision of 0.93, which underlines its ability to perform well regardless of the chosen level of certainty. This flexibility is important in practical applications where the environment and the variability of the datasets could affect the outcomes of the models [7]. Furthermore, the work also provides a computationally efficient and scalable solution for large-scale applications that is well illustrated by the relatively stable validation accuracy of our model at epoch 10 of 96.94% and validation loss of 0.3302. These results also imply potential for applying our model to large scale drone imagery data sets without much compromise on performance.

Even when the results are framed within the context of the existing literature on using drones for tree enumeration, it can be noted that our study adds an important piece to the puzzle. In contrast to prior works that utilized LiDAR-based segmentation [4,5] or only aimed at enhancing classification accuracy [1,2], the addition of a detection head expands the capabilities of VGG16 for real-time aerial image analysis. This enhancement not only enables better tree counting but also creates a basis for wildlife recording, city planning, and agricultural use, where accuracy and coverage are essential [8,9]. Thus, our results support the use of this method, which fills gaps in current approaches and establishes new best practices for the field.

## Conclusion

This study demonstrates the successful development and evaluation of the Aerial Eye Tree Detection Algorithm (AETDA) for classifying and localizing tree types—banana, oil palm, and coconut trees—in high-resolution aerial imagery. By comparing multiple deep learning architectures (ResNet, VGG16 with Detection Head, DETR, and YOLOv8), it was evident that YOLOv8 outperformed other models in terms of precision, efficiency, and multi-object detection accuracy, making it the optimal choice for this task.

Modified with a custom detection head, the VGG16 model achieved high classification accuracy: 98% of the training dataset. VGG16 has no inherent object detection functionality; therefore, a detection head was, for the first time, integrated to enhance its detection functionality. This novel integration enabled VGG16 not only to categorize tree types but also to predict the coordinates of the bounding boxes for the object detection within the image. However, incorporating the detection head with VGG16 provided a solution for detecting multiple tree types with less accuracy in bounding box precision compared to YOLOv8. Still, the high classification capability of this architecture proved that this architecture is useful for the detection of tree types in the dataset. This is the adaptation that is new to this paper; while the VGG16 has some shortcomings in the object localization aspect, it makes this network very useful for tasks that involve both classification and localization of objects.

ResNet, used primarily for feature extraction, provided strong classification performance with an accuracy above 90%. However, ResNet was inherently limited to single-instance predictions within an image and lacked the ability to produce bounding boxes. Its performance in identifying more than one tree in an image was restricted, making it suboptimal for complex multi-object detection tasks like ours. Although it provided reasonably accurate classifications for single trees, it could not meet the project’s multi-instance localization requirements.

The Detection Transformer (DETR), with its transformer-based architecture, was evaluated for its strength in spatial relationship detection and multi-object classification. While DETR demonstrated excellent tree counting accuracy and reliable classification performance, it faced challenges with precise localization. Bounding boxes often overlapped significantly, impacting the quality of object separation, and while it effectively recognized tree types, the bounding box accuracy was inconsistent, preventing it from delivering the high-precision annotations necessary for this study. Nonetheless, DETR’s almost-perfect counting ability was noteworthy, indicating its potential for specific applications where object counting is a primary focus.

Given the challenges observed with these models, YOLOv8 emerged as the most effective solution. As the latest version of the YOLO architecture, YOLOv8 integrates anchor boxes and Non-Maximum Suppression (NMS) techniques to enhance localization precision and reduce bounding box redundancy. It performed highly accurate multi-object detection with reliable bounding boxes, meeting the project’s requirements for high precision in both localization and classification. Moreover, its lightweight design ensured efficient processing, enabling real-time inference with marginal computational demands.

Overall, the results of this project suggest that VGG16 with a detection head as well as YOLOv8 models is a robust and efficient solution for automated tree detection and classification, excelling in both accuracy and efficiency across diverse environments and observational perspectives. With its potential applications in forestry management, agricultural monitoring, and environmental conservation, this approach offers a scalable solution for mapping and tracking tree distributions with high accuracy, supporting critical ecological and agricultural insights.
